# Optical OFDM Error Floor Estimation by Means of OTDR Enhanced by Front-End Optical Preamplifier

**DOI:** 10.3390/s21217303

**Published:** 2021-11-02

**Authors:** Adriana Lipovac, Vlatko Lipovac, Mirza Hamza, Vedran Batoš

**Affiliations:** 1 Department of Electrical Engineering and Computing, University of Dubrovnik, 20000 Dubrovnik, Croatia; vlatko.lipovac@unidu.hr (V.L.); vedran.batos@unidu.hr (V.B.); 2 Department of Telecommunications, Faculty of Electrical Engineering, 71000 Sarajevo, Bosnia and Herzegovina; mhamza@etf.unsa.ba

**Keywords:** OTDR, BER floor, dynamic range, optical amplifier

## Abstract

Optical time-domain reflectometer (OTDR) enables simple identification and localization of a plethora of refractive and reflective events on a fiber link, including splices, connectors and breaks, and measuring insertion/return loss. Specifically, large enough OTDR dynamic range (DR) and thus high signal-to-noise-ratio (SNR) enable clear far-end visibility of longer fibers. We point out here that, under such conditions, the optical bit-error-rate (BER) floor is dominantly determined by reflective events that introduce significant return loss. This complements the OTDR legacy tests by appropriate optical BER floor estimation in the field. As high SNR implies inter-symbol interference as dominating error generating mechanism, we could apply the classical time-dispersion channel model for the optical BER floor determined by the root-mean-square (rms) delay spread of the actual fiber channel power-delay profile. However, as the high-SNR condition is not always fulfilled mostly due to insufficient DR, we propose here inserting a low-noise optical preamplifier as the OTDR front-end to reduce noise floor and amplify the backscattered signal. In order to verify the model for the exemplar test situation, we measured BER on the same fiber link to find very good matching between the measured BER floor values and the ones predicted from the OTDR trace.

## 1. Introduction

**Figure 1:**
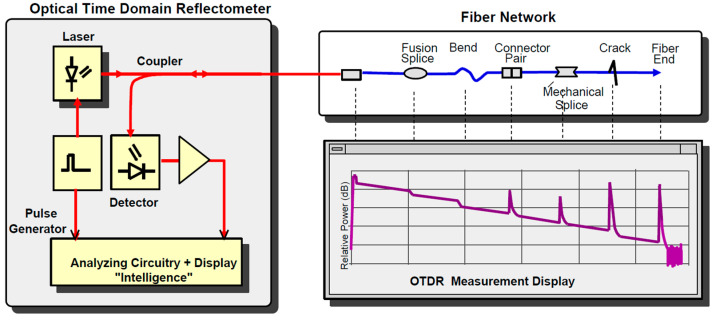
Optical time-domain reflectometer (OTDR) architecture and trace.

Considering test and measurement equipment used during installation and maintenance of contemporary lightwave transmission systems, optical time-domain reflectometer (OTDR) is still an outstanding fault detection and localization tool enabling simple and integral characterisation of a variety of refractive and reflective events on a fiber link, such as splices, connectors and breaks, with according measurements of insertion/return loss [1,2,3,4], Figure 1.

Basically, OTDR transmits a high-power laser light pulse into the fiber, and detects reflections from various structural irregularities along the fiber (backscatter), describing them in the power-loss-versus-distance format, i.e., expressing the time axis in distance units by inherently taking into account the speed of light in the fiber [5,6,7,8].

The main functional OTDR entities are: microprocessor, pulse trigger and generator, laser diode, optical directional coupler (ODC), detector, analog-to-digital converter (ADC) and display, Figure 1.

**Figure 2:**
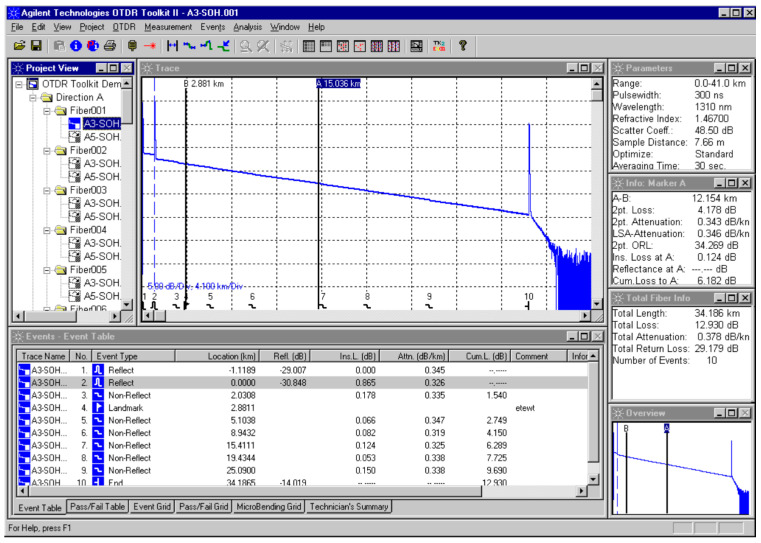
Typical OTDR trace.

The test is initiated by the microprocessor executing the according instructions and sending the control signals to the pulse generator to trigger the laser. The outgoing series of laser light pulses transverses the ODC on their way into the fiber under test, whereas the reverse backscatter incoming signal is channeled by the ODC towards the detector—most often the avalanche photodiode (APD). Further on, the received signal proceeds through the ADC to the microprocessor for final analysis and display, which specifically includes statistical averaging to improve the signal-to-noise ratio (SNR), and thus enhance the OTDR trace by displaying average power of a number of sampling points, rather than the instantaneous values, Figure 2.

**Figure 3:**
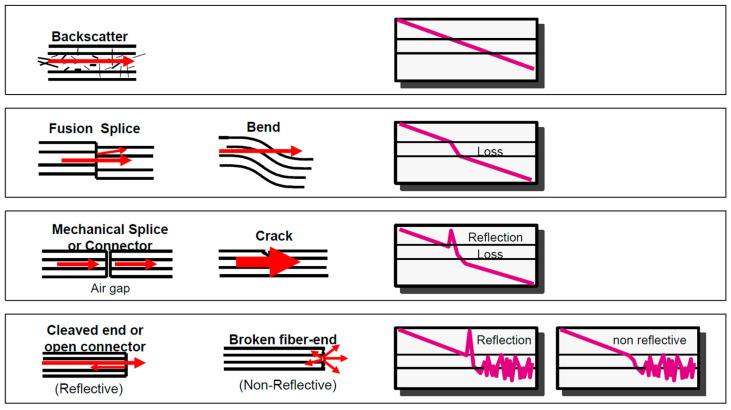
Various OTDR trace events’ signatures.

Actually, the small-amplitude backscatter signal returning to the OTDR includes Rayleigh scattering and Fresnel reflections as well. The Rayleigh scattering enables calculating the fiber attenuation as a function of distance, which is represented by the constantly falling part of an OTDR trace, whereas Fresnel reflection causes a strong reflection back, and so enables detection of physical events along the fiber link identifiable by spikes in OTDR trace due to connectors, mechanical splices, fiber breaks or opened connectors, Figure 3.

Non-reflective events-fusion splices and bending, and are visible as discrete drops in backscatter level, as these cause loss, but no reflection, whereas mechanical splices, connectors and cracks cause both reflection and loss.

The events’ signatures are presented in Figure 3.

Specifically, the OTDR dynamic range, defined as the difference (in dBs) between the incoming reflected backscatter signal level at the OTDR front end and the noise floor level at the far end of the fiber, also taking into account attenuation introduced by connectors, splices and splitters, determines the far-end events visibility and measurement accuracy, especially for a long-fiber. Consequently, the dynamic range should be about 5 to 8 dB larger than the maximal fiber loss measurable by the OTDR [1].

Moreover, considering the dynamic range from the bottom up perspective, the low limit of the OTDR dynamic range, i.e., the noise floor is commonly referred to as the signal value for which the SNR equals unity, observed with the longest pulse (i.e., the strongest) and the three-minutes long averaging [9].

On the other hand, in R&D, production, installation and advanced maintenance of fiber transmission systems, their end-to-end performance is mostly expressed by the bit-error-rate (BER), where specifically the residual BER (BER floor), is the ultimate irreducible lower-limit performance metrics for high-SNR conditions, when time dispersion (and consequent inter-symbol interference (ISI) remains the dominant error generating mechanism), and it is measured out-of-service, using the pseudo-random binary sequence (PRBS) generator and receiver.

Most often, single BER tester (BERT) comprising both the PRBS generator and the receiver, is used whereas the loop-back is made at the far end of the fiber.

**Figure 4:**
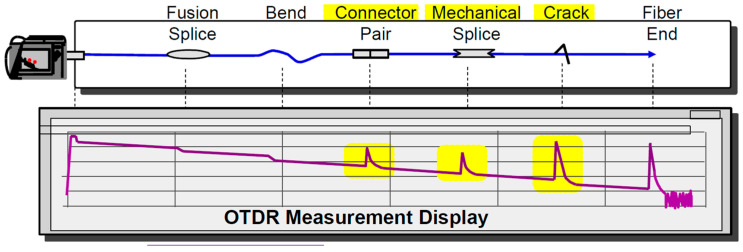
Dominant reflective events identified by OTDR.

Now, back to OTDR trace, if it exhibits enough large dynamic range and, consequently, large SNR, the fiber transmission performance—effectively the BER floor, can be regarded as being dominantly determined by fiber time dispersion, i.e., by the OTDR-identified reflective events along the fiber, Figure 4.

So, the question of interest here is: can we (reliably) estimate the fiber BER floor from the related OTDR trace in this case?

With this respect, still some (mostly older) OTDR units cannot fulfill the above-elaborated condition for residual BER estimation, due to their insufficient dynamic range.

Therefore, in this paper, we propose a simple and cost effective solution to extend the OTDR dynamic range for given laser saturation energy determined by the chosen pulse width, which is lowering the OTDR noise floor. This makes sense, as the OTDR receiver noise figure is, to a large extent, determined by the (large) one of the ODC (as of the passive device) [3]—the very first block which the returning backscatter passes through before entering the detector.

Thus, we inserted a low-noise high-gain optical preamplifier as the OTDR front end, justifiably expecting that the noise figure of the series would be dominantly determined by the (lower) noise figure of such a front end, which, according to our preliminary tests in the framework of a network operator environment, enabled larger OTDR dynamic range and thus SNR, where the latter is a prerequisite for reliable OTDR aided BER floor estimation.

In Section 2, we review the relevant OTDR basic analytical model to relate the according residual BER expression to the crucial time dispersion parameter—root-mean-square (rms) delay spread of the optical channel delay profile that we consider conforming to the OTDR trace. Then, we review the model for OTDR dynamic range extension by inserting the low-noise high-gain optical amplifier (OA) as the external front end, and thus fulfil the condition for estimating the residual BER.

In Section 3, we provide the according analysis to qualify matching between the OTDR-based and directly measured BER floor values.

We present and discuss the preliminary test results, and then summarize the conclusions in Section 4.

## 2. Analysis

On the other hand, the orthogonal frequency-division multiplexing (OFDM) has been more and more used for long-haul optical transmission [1,2,3], where the real-time digital signal processing has enabled signal rates to surpass 10 Gbit/s, with high spectral efficiency and flexible network design.

### 2.1. OFDM BER Floor Model

As it is challenging to uniquely relate the OTDR trace events (primarily the reflective ones) to the parameter(s) determining the OFDM BER error floor under high-SNR conditions and therefore dominant time dispersion, which implies that we can apply the m-subcarrier OFDM BER floor model [10], specifically when sampling time is just upon the arrival of the first impulse of the channel impulse response [2].

So, we consider the power-delay profile as the sum of *N* impulses with powers $A_{i}^{2}$, and delays $\tau_{i}$, i=1,2,…N [10].

Then the BER floor is:

(1) $$BER\approx \frac{k_{MOD}}{2\sqrt{\pi \cdot m}}\cdot \frac{\sqrt{E[\tau^{2}]}}{T_{s}}=\frac{k_{MOD}}{2\sqrt{\pi \cdot m}}\cdot \frac{\sqrt{\sum_{i=1}^{N}A_{i}^{2}\tau_{i}^{2}}}{T_{s}}$$

where $\frac{\sqrt{E[\tau^{2}]}}{T_{s}}$ is the standard rms delay spread (normalized to the symbol time *T*_S_) of the channel delay power profile (describing time dispersion), whereas the modulation coefficient $k_{\mathrm{MOD}}$, is given by the ratio of the symbol error rate (SER) for the M-QAM modulation to BER:

(2) $$k_{\mathrm{MOD}}=\frac{\frac{SER_{M\mathrm{QAM}}}{BER_{\mathrm{BPSK}}}}{ldM}=\{\begin{array}{c} \;\;2/2=1;\;4\mathrm{QAM}\\ \;3/4=0.75;\;16\mathrm{QAM}\\ 3.5/6=0.583;\;64\mathrm{QAM} \end{array}$$

However, in this paper, with no loss in (OFDM) generality, we consider simple binary transmission, and therefore adopted that $k_{\mathrm{MOD}}=1$, so that (1) simplifies to:

(3) $$BER\;\approx \;0.28\;⸱\;\frac{\sqrt{E[\tau^{2}]}}{T_{s}}$$

Furthermore, as for the contemporary transmission networks including not only fiber links but the microwave ones, too, the bit-oriented transmission performance standards have long ago been replaced with the data block-oriented ones [10], let us now derive the Block Error Ratio (BLER) from BER estimated by (3) [10,11]:

We can justifiably assume that, although the common binomial distribution statistically well describes mutually independent bit error occurrences within an *L*-bits-long data block, in this case, we consider that the appropriate error generating model should still preserve (moderate) mutual dependability among the individual bit error occurrences. This conforms to the statistical model of sampling without replacement, well described by the hypergeometric distribution of errors within an erroneous data block (containing one or more erroneous bits), which provides the following *BLER* vs. *BER* relationship [10,11]:

(4) $$BLER\left(BER\right)\approx 1-{\left(1-BER\right)}^{L}\;$$

### 2.2. OTDR Trace as Power-Delay Profile

Now the task is to estimate BER floor from the normalized rms delay spread $\frac{\sqrt{E[\tau^{2}]}}{T_{s}}$ of the fiber channel power-delay profile *P*_BS_(*z*), which we consider to conform to the OTDR backscatter trace (as describing time dispersion alike).

At the observation point that is *z* apart from the OTDR, the fiber response on the pulse (of amplitude) *P*_T_^1/2^(*z*), is its convolution with the fiber impulse response *h*(*τ*) of an OTDR system, where:

*P*_BS_^1/2^(*τ*) = *P*_T_^1/2^(*τ*) * *h*(*τ*); *τ* = (2*z ⸱ n*)/*c* (5)

So, to obtain the impulse response *h*(*τ*), we need to de-convolve it from the OTDR transmitted pulse *P*_T_^1/2^(*τ*), i.e., solve (5) per *h*(*τ*), which leads to [12]:

*h*(*τ*) = c/2*n⸱E*_i_*⸱S⸱η*_i_*⸱*exp(−2*αz*) (6)

where *c*, *n*, *E*_i_ and *η*_i_ are speed of light, laser energy, fiber average refractive index, and quantum efficiency of the avalanche photodiode detector, respectively, whereas *S* < 1 denotes the efficiency to confine the incident light (so only a certain part of the scattered light travels back to the OTDR), and *α* stands for fiber loss.

However, practically, for large dynamic range, we could consider the OTDR pulse (usually about 1 µs long) to be still short enough (with respect to the reciprocal OTDR receiver bandwidth)—almost as an impulse described by delta function:

*P*_T_(*z*) = *δ*(−2*αz*) (7)

so that (5) modifies to:

*P*_BS_^1/2^(*z*) = *δ*(−2*αz*) _*_h(*z*) = *h*(*z*); *z* = *c* · *τ*/2 (8)

This implies that the normalized power—delay profile of the fiber can be approximated by the normalized (to unity area) relative OTDR trace as [12,13]:

׀*h*(*τ*׀^2^ = *P*_BS_(*z* = *c* · *τ*/2) (9)

### 2.3. OTDR Dynamic Range

As mentioned above, the dynamic range between the maximal backscattered power *P*_BS_ (0) at the input of the fiber, and the effective noise equivalent power *NEP*_eff_—the noise floor where SNR = 1 [9], can be presented as:

*DR* [dB] = [*P*_BS_ (0) − *NEP*_eff_] (10)

The residual BER can be accurately estimated only by OTDR of sufficient *DR* with enough margin against attenuation of not only the actual fiber, but also of connectors, splices and splitters that the testing OTDR encompasses.

**Figure 5:**
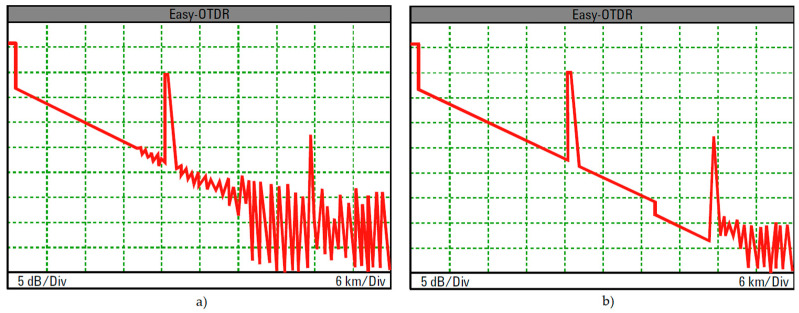
OTDR trace; (**a**) small dynamic range, (**b**) large dynamic range.

**Figure 6:**
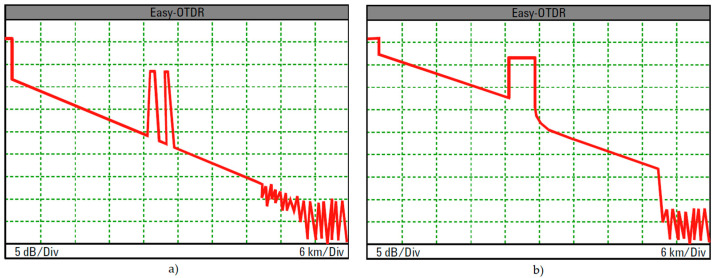
OTDR trace; (**a**) with short pulse for better event resolution; (**b**) with longer pulse for larger dynamic range.

The high-SNR condition fulfilled with large enough OTDR dynamic range, is basically achieved with long enough transmitted pulses, Figure 5, but these also worsen the event resolution and enlarge the attenuation dead zone, Figure 6.

Moreover, an OTDR trace being effectively an attenuated two-way squared response of the fiber link to the transmitted laser pulse, can only be considered the power-delay profile for a short enough laser pulse which is realistically considerable as impulse.

In addition to pulse width, receiver bandwidth is another crucial parameter, which determines the actual OTDR resolution, as for short transmitted pulses, the OTDR receiver (photo-detector) bandwidth should be large enough, at least 40 % wider than the reciprocal pulse width [12,13].

On the other hand, from the bottom-up perspective, with already chosen pulse width, dynamic range can be increased by lowering its lower bound *NEP*_eff_, with visual outcome similar to the one illustrated in Figure 5.

### 2.4. OTDR Receiver Noise Floor Reduction

Let us express *NEP*_eff_ of a receiver cascade of blocks by the more common noise figure *F* relating the (shot) noise power spectral density *P*_no_ at the receiver output to the input noise power spectral density *P*_ni_ multiplied by the receiver gain *G* [12]:

*F* = *P*_no_/*G* ·*P*_ni_ = 1 + *NEP*_eff_/*G* · *P*_ni_ (11)

Thus, (11) expresses linearity of the noise floor *NEP*_eff_ with the noise figure *F* [12]:

*NEP*_eff_ = (*F* − 1) *G* · *P*_ni_ (12)

#### 2.4.1. Noise Figure of ODC Coupled OTDR

As it can be seen in Figure 1, the OTDR backscatter firstly transverses the ODC and then the APD detector of the OTDR receiver, where the ODC’s noise figure (as of a passive device) equals its intrinsic insertion loss [3].

Furthermore, as, generally, the noise figure *F*_1,2_ of the cascaded two blocks is, to the large extent, determined by the noise figure *F*_1_ of the first block (with large gain *G*_1_ >> 1) in the series:

*F*_1,2_*=F*_1_ + (*F*_2_ − 1)/*G*_1_ ≈ *F*_1_ (13)

the above classic relation shows that *F*_2_ (unless it is very large), does not contribute significantly to *F*_1,2_ characterizing the whole cascade [12].

However, in case of a noise-rich (older-generation) OTDR receiver’s APD [1], the passive front-end ODC with insertion loss *A*_DC_ (equal to its own NF), deteriorates the noise figure (13) of the cascade even further to [12]:

*F*_OTDR_*=A*_DC_ + (*F*_APD_ − 1) · *A*_DC_ = *A*_DC_ · *F*_APD_ (14)

Equation (14), implies that, for wide class of OTDRs, we can justifiably consider the receiver noise figure, and thereby the noise floor, relatively high.

On the contrary, state-of-the-art erbium-doped fiber amplifiers (EDFA) provide high and flat gain often in the range of 30 dB, as well as low noise figure below 4 dB [13,14], which make them useful as OTDR external pre-amplifiers that could enable high-SNR BER testing [12,14,15,16,17,18,19,20,21].

Moreover, a low-insertion-loss passive optical circulator (OC) is an appropriate device for signal separation needed for connecting an EDFA to the OTDR [3,12].

#### 2.4.2. OA/OC/OTDR Dynamic Range Prediction Model

From (13), analogously to (14), the noise figure of the whole cascade consisting of the 4-port OC, OA and OTDR, is [12]:

*F*_OC+OA+OTDR_*=F*_OC+OA_ + (*F*_OTDR_ − 1)/*G*_OC+OA_ ≈ *F*_OC_ (15)

where we considered *G*_OC+OA_ ≈ *G*_OA_ >> *A*_OC_ (quite justifiably as *G*_OA_ is of the order of 10^3^ and thereby much larger than the OC insertion loss *A*_OC_).

Thereby, recalling (14), from (15) it is obvious that [12]:

*F*_OC+OA+OTDR_ ≈ *A*_OC_ ·*F*_OA_ << *F*_OTDR_
*=*
*A*_DC_ · *F*_APD_ (16)

i.e., that introducing the OTDR front-end optical pre-amplifier significantly reduces the noise figure, and thereby widens the dynamic range Δ*DR* as it implies from (10):

Δ*DR* = [*P*_BS_(0) − *NEP*_OC+OA+OTDR_] − [*P*_BS_(0) − *NEP*_OTDR_] *= NEP*_OTDR_ − *NEP*_OC+OA+OTDR_ (17)

From (12), it is obvious that, for certain input noise power *P*_ni_, the noise floor *NEP*_OTDR_ for the OTDR alone is:

*NEP*_OTDR_ = (*F*_OTDR_ − 1) · *G*_OTDR_ (18)

whereas for the cascaded OC, OA and OTDR, it is:

*NEP*_OC+OA+OTDR_ ≈ *NEP*_OA_ = (*F*_OA_ − 1) · *G*_OA_ · *P*_ni_ (19)

So, the noise floor reduction (and consequent dynamic range extension) as a benefit of inserting the preamplifier, can be obtained if we divide (18) by (19), or make the according subtraction in dBs, which finally provides the following prediction [12]:

Δ*DR* = (*P*_sOA+OTDR_ /*P*_nOA+OTDR_)/1 = *G*_OA_ ⸱*NEP*_OTDR_/(*G*_OTDR_⸱*NEP*_OA_+*NEP*_OTDR_) (20)

By adopting common values for the relevant parameters of interest, specifically, the *NEP*_OTDR_ to be close to −50 dBm, *G*_OA_ of 30 dB and *F*_OA_ of 6 dB, resulting with *NEP*_OA_ = 447 × 10^−5^ nW, whereas considering *G*_OTDR_ = 10 dB, (20) provides the predicted dynamic range extension value of Δ*DR* ≈ 30 dB [12].

Thereby, the high-SNR condition for accurate BER floor estimation is fulfilled with large credibility.

## 3. Test Results

### 3.1. Test System

We used the HP 8147A OTDR, designed for fiber characterization by network operators for installation and field maintenance of their optical transmission systems [9].

**Figure 7:**
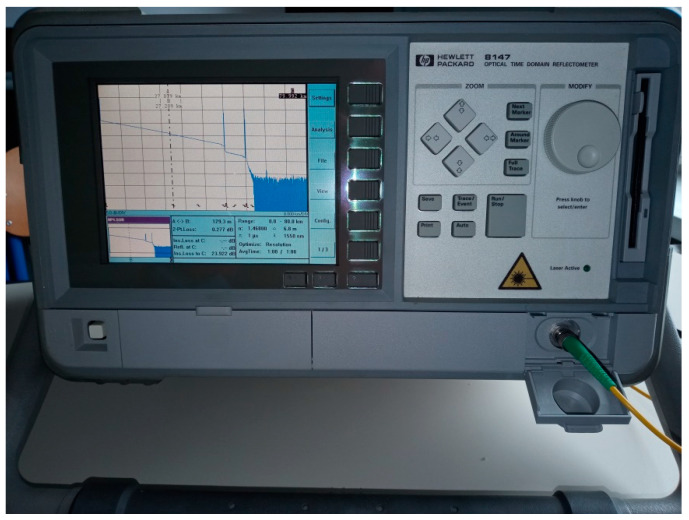
OTDR under test.

**Figure 8:**
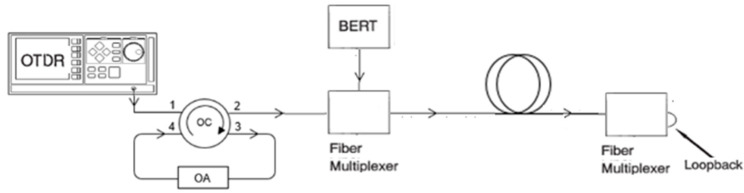
OTDR and the two-way BER test configuration.

The OTDR in Figure 7 was used with the 1310/1550 nm plug-in optical interface module, and it was connected in the test configuration alternately with the BER tester (BERT) according to Figure 8, where it can be seen that the OC port 2 is isolated from the adjacent OC port 3, as these are externally interconnected by the OA.

We used a standalone Ethernet tester to detect and count erroneous frames of 1518 Bytes, each containing at least a single bit error. In this work, we consider the frame error rate as BLER, and adopted the operation with maximal possible throughput while performing the out-of-service end-to-end Ethernet measurements according to Rec. RFC 2544 [1], with the SW loopback at the far-end.

As network operators prefer in-service practical test methods, which include fiber testing with the OTDR, as well as the end-to-end transmission performance testing with the BERT, we used both of these connected to a Gbit/s “dark” fiber, to not interrupt active fibers carrying live traffic by neither OTDR nor BER testing (The end-to-end connection encompasses both fiber and microwave radio-relay sections [22]).

As our BER floor model requires high SNR and thus large DR, both earlier considered as achievable by longer transmitted pulses (at the price of being less realistically considerable as impulses), we selected 1 µs as a good compromise for the fiber under test.

### 3.2. Preliminary Test Results

**Figure 9:**
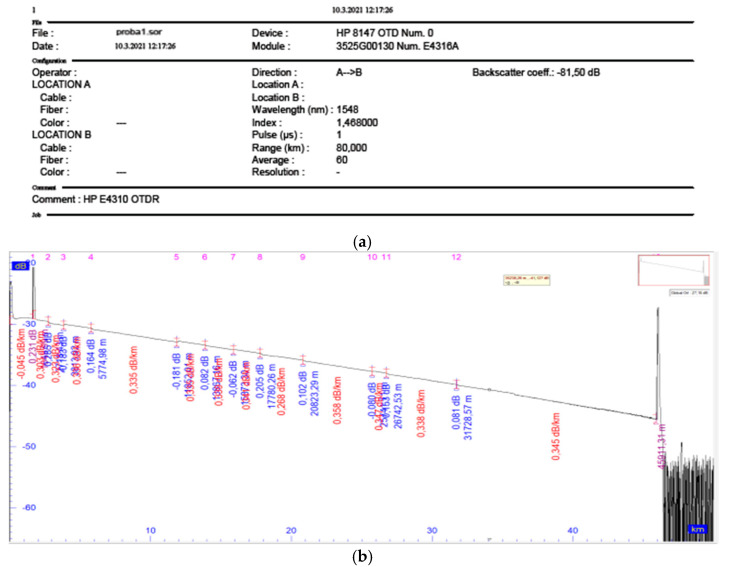
OTDR tests: (**a**) setup; (**b**) trace printout with discovered and qualified events (with distance [km], and power level [dB], written in blue font, and fiber loss [dB/km], in red one).

Our preliminary test results are aimed to just verify the proposed concept, whereas the follow-up high-accuracy tests of this kind can be repeated as many times as needed. The exemplar OTDR setup screen shot and a trace with discovered and qualified events, are presented in Figure 9, followed by Table 1 and Table 2 displaying the BER/BLER test results estimated by the proposed model (and indexed by “T.DISP“ which stands for time dispersion, i.e., for large SNR), as well as the test results obtained by means of the BER tester.

Moreover, the below tables also contain the BER/BLER entries (indexed by “ABSTR“) as the overpesimistic “worst-case scenario“ values obtained by applying the well-known BER expression for the M-QAM signal transmission over the additive white Gaussian noise (AWGN) channel, as a function of the ratio of the energy *E*_b_ of a bit to noise spectral density *N*_0_, is [10]:

(21) $$BER=\frac{4\cdot Q\left(\sqrt{\frac{3\frac{E_{b}}{N_{0}}\cdot \log_{2}M}{M-1}}\right)}{\log_{2}M}=4\cdot Q\left(\sqrt{3\frac{E_{b}}{N_{0}}}\right)$$

where *Q* denotes the Gaussian tail function.

From the OTDR trace, the actual *SNR* (determining *E*_b_/*N*_0_) value at the far end, is read and substituted into (21) abstracting the actual BER by the AWGN-only based one (as a sum of a number of mutually independent and even non-Gaussian small distortions is subject to the application of Central Limit Theorem and can therefore be considered to have Gaussian distribution and the according mean and variance [10].

Then, we can use such an AWGN-modelled BER/BLER as another reference (in addition to the BER testing) for verification of the proposed BER/BLER predictions.

As it is evident in Table 1 and Table 2, there is good matching between the OTDR-aided residual BER and BLER estimations with the corresponding actually measured values, respectively, both with and without optical preamplifier (whose benefit for reducing BER and BLER was minor here due to relatively large SNR in both cases).

Furthermore, in the above tables it is noticeable that the AWGN-abstracted BER and BLER are somewhat larger than the corresponding values obtained by both the proposed model and real life testing, with or without the preamplifier applied. This can be interpreted as the consequence of worst-case design of the AWGN abstraction (coming out of wide-sense stationary uncorrelated scattering (WSS-US) assumption, when no dominant reflective events are identified on the OTDR trace.

## 4. Conclusions

A simple and cost effective means for estimating the fiber optic link BER and BLER floor from an OTDR trace, is proposed to extend OTDR usability during installation and maintenance of fiber optic links, beyond bare identifying and visualizing various error-generating impairments (events), to encompass predicting the end-to-end transmission performance.

With this regard, we considered the OTDR-identified reflective events as determining the standard time-dispersion indicator—mean delay spread of the power-delay profile, which is known to determine the BER floor (and thus BLER, too).

Moreover, for the case when the high-SNR condition is not fulfilled for estimating the residual BER, we proposed the OTDR dynamic range extension by introducing a low-noise high-gain EDFA optical preamplifier in front of the OTDR, to reduce the noise floor of the cascade, and thus increase SNR.

The theoretical model was preliminary validated experimentally, showing good matching between the OTDR-predicted and actually measured residual BER, for short transmitted pulses and large enough OTDR receiver bandwidth.

This work was aimed to model, quantify, and verify predicting fiber OFDM transmission performance from OTDR trace, and so pave the way to high-accuracy measurements that can be repeated as many times as needed within professional R&D and field test campaigns, taking into account design and deployment issues as well, and using sophisticated hardware and industry-standard software simulation tools.

## Figures and Tables

**Table 1:** OTDR trace-based estimate vs. measured bit-error-rate (BER) floor; 50 and 80 km fiber link, 1 Gbit/s.

Link Length	50 km	80 km
BER_OTDR_T.DISP_	4.13 × 10^−12^	6.31 × 10^−12^
BER_OTDR_T.DISP_/OA	4.08 × 10^−12^	6.22 × 10^−12^
BER_OTDR_ABSTR_	4.84 × 10^−12^	6.97 × 10^−12^
BER_OTDR_ABSTR_/OA	4.77 × 10^−12^	6.83 × 10^−12^
BER_BERT	4.49 × 10^−12^	6.89 × 10^−12^
BER_BERT/OA	4.46 × 10^−12^	6.7 × 10^−12^

**Table 2:** OTDR trace-based estimate vs. measured BLER floor; 50 and 80 km fiber link, 1 Gbit/s.

Link Length	50 km	80 km
BER_OTDR_T.DISP_	5.02 × 10^−8^	7.63 × 10^−8^
BER_OTDR_T.DISP_/OA	4.96 × 10^−8^	7.32 × 10^−8^
BER_OTDR_ABSTR_	5.52 × 10^−8^	8.42 × 10^−8^
BER_OTDR_ABSTR_/OA	5.48 × 10^−8^	8.24 × 10^−8^
BER_BERT	5.45 × 10^−8^	8.37 × 10^−8^
BER_BERT/OA	5.42 × 10^−8^	8.14 × 10^−8^

## Data Availability

Not applicable.
